# Respiratory Syncytial Virus in a Child With Dravet Syndrome: A Case Report

**DOI:** 10.7759/cureus.59405

**Published:** 2024-04-30

**Authors:** Nga N Tran, James Liu, Tyler Bullock, David Flowers

**Affiliations:** 1 Simulation Center, Edward Via College of Osteopathic Medicine, Auburn, USA; 2 Medicine, Kirksville College of Osteopathic Medicine, Kirksville, USA; 3 Nursing, Columbus State University, Columbus, USA; 4 Pediatrics, Piedmont Columbus Hospital, Columbus, USA

**Keywords:** fever, dehydration, respiratory syncytial virus, seizures, dravet syndrome

## Abstract

The objective of this case report is to describe and document a case of respiratory syncytial virus (RSV) in a pediatric patient with Dravet syndrome (DS), also known as severe myoclonic epilepsy of infancy. Febrile seizures are often a complication in a patient with DS and can lead to status epilepticus, necessitating measures to prevent triggers such as fever, electrolyte imbalance, or dehydration. An increased awareness and understanding of DS can facilitate the identification of warning signs.

A two-year-old female with a past medical history of DS with focal and generalized features presented to the pediatric emergency department (ED) with a five-day history of cough, fever, and decreased oral intake. The patient’s parents accompanied her and expressed concerns regarding the risk of seizures associated with a rise in body temperature, as they had been alternating between acetaminophen and ibuprofen to manage her fever with a maximum recorded temperature of 101.5℉. She exhibited signs of increased work of breathing, necessitating the administration of supplemental oxygen via nasal cannula. Blood samples were obtained and resulted in the development of metabolic acidosis. A respiratory panel confirmed the presence of an RSV infection, promoting the administration of breathing treatment with albuterol and ipratropium bromide. The patient was admitted for dehydration and was started on ½ normal saline/potassium chloride 20 mEq at 40 mL/hr. Additionally, her home medication regimen was resumed to minimize the risk of seizures. Given the patient’s complications and increased risk of seizure, she was transferred to higher-level care where her status improved after the placement of a percutaneous endoscopic gastrostomy (PEG).

This case underscores the complexities involved in managing patients with DS, particularly when complicated by respiratory illness and electrolyte imbalances that can lower the seizure threshold. This patient received a combination of diet and medications to prevent seizures, as well as allow for recovery and correction of the underlying metabolic acidosis. The transfer to a higher level of care in this case was necessary to allow for the specialized resources and expertise needed to handle this case.

## Introduction

Dravet syndrome (DS) is a rare type of epilepsy in which patients typically present with a seizure during the first year of life with a varying degree of cognitive delay [1]. Approximately 85% of DS patients have a mutation in the *SCN1A* gene [2]. In most cases, the SCN1A mutation arises spontaneously through a mosaic de novo mutation from unaffected parents [3]. Only in very rare cases is the SCN1A mutation variant passed from parents to children [4]. Although genetic testing is not required for the diagnosis of DS, it plays a crucial role in effectively managing a patient's symptoms. It is worth noting that a small percentage of individuals who exhibit recurrent seizures and epilepsy before the age of one year, who do not possess the SCN1A gene mutation, may have SCN1A exonic deletions, or chromosomal arrangements, or the mutation of protocadherin 19 (PCDH19), causing similar symptoms [5]. Further studies are needed to explore the pathogenesis of the remaining 20% of DS cases which do not align with the SCN1A gene mutation. While elevated body temperature is a prominent trigger for DS episodes, it is important to acknowledge that the definitive diagnosis of DS is two or more seizures, with or without fever, before the age of one year [6].

Seizures can be triggered or exacerbated by mild elevation in body temperature, leading to potential complications such as aspiration, injury, and even sudden unexpected death in epilepsy (SUDEP). In some cases, patients with Dravet syndrome may exhibit resistance to anti-epileptic medications. However, studies have shown that a ketogenic diet is an effective first-line therapy and is not inferior to anti-seizure medications [7]. While adverse effects have been reported on the gastrointestinal system and for patients with metabolic disorders, including weight loss, a ketogenic diet is generally considered safe [7]. The ketogenic diet puts the body into starvation and helps produce antioxidants, which are thought to reduce the seizure threshold [7].

RSV infection presents with symptoms similar to a common cold, including but not limited to fever, cough, and rhinorrhea. Understanding the significance of RSV in investigating DS is essential due to its capacity to induce fever, ultimately triggering epileptic seizures in individuals with DS [4]. Symptomatic management of RSV involves the use of pain relievers such as acetaminophen and ibuprofen to control the patient’s temperature [8].

The management of DS requires early diagnosis, allowing for effective prevention of triggers. This includes prophylactic seizure medications to lower the risk of sudden unexpected death in epilepsy (SUDEP) [9]. In patients with pharmacoresistant DS, stiripentol, cannabidiol, and fenfluramine are good alternatives for preventing fatal outcomes [9]. It is vital to recognize the early signs of febrile seizures, as those who do have DS have an increased 15-20% mortality risk due to SUDEP [10].

## Case presentation

A two-year-old female with a past medical history of Dravet syndrome (DS) and epilepsy, characterized by both focal and generalized features, presented to the pediatric emergency department by her parents. The patient had been experiencing symptoms of cough, rhinorrhea, congestion, fever with a maximum temperature of 101.5℉, and decreased oral intake for 5 days. In an effort to reduce the fever, her parents had been alternating between over-the-counter acetaminophen 160 mg and ibuprofen 100 mg but were concerned about the heightened risk of seizures. The patient was on a ketogenic diet, as recommended by her neurologist, to help manage seizures, but had been experiencing limited solid and liquid intake over the last five days.

The patient was adopted at the age of four months by her grandparents following the passing of her biological mother due to an overdose. The patient receives regular follow-up care from a neurologist to monitor her seizure activity. Her first seizure was in October 2020, triggered by fevers, and her last grand mal seizure occurred four months prior to her presentation in the pediatric emergency room and prompted the initiation of a ketogenic diet. The patient’s medication regimen included cannabidiol, fenfluramine, levocarnitine, midazolam, stiripentol, and clobazam.

**Figure 1:**
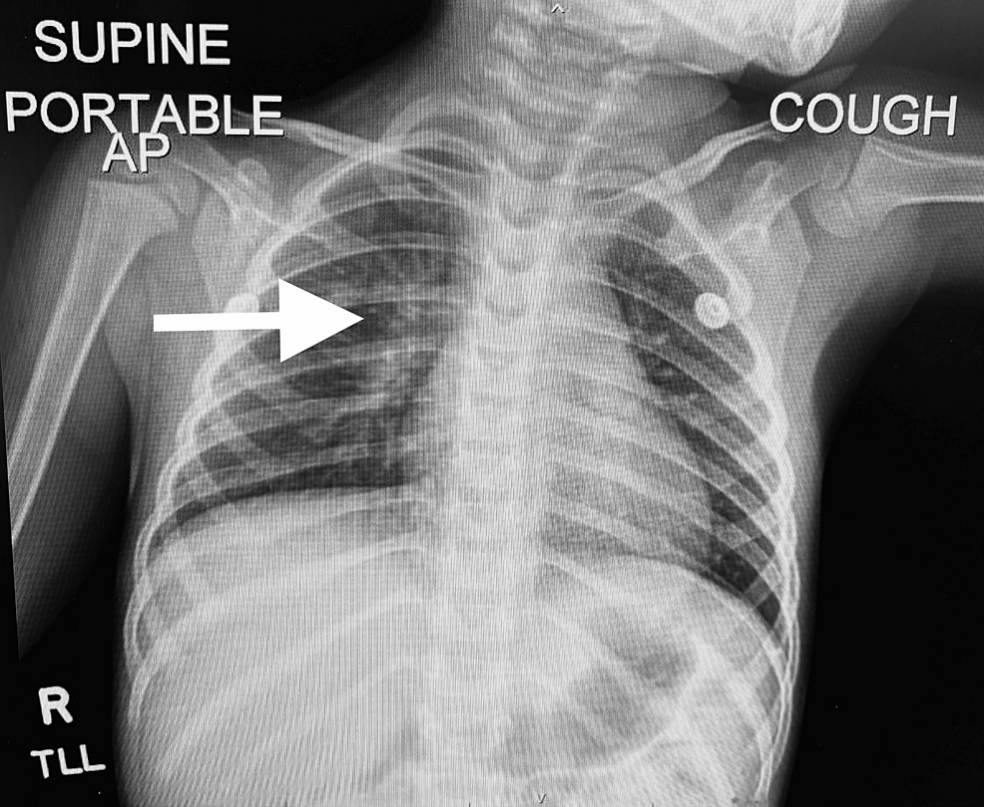
Chest X-ray of the patient at arrival Chest X-ray demonstrates minimal patchy airspace opacity in the right parahilar region

Upon arrival, her blood pressure was 92/58 and her temperature was 100.3℉. Her physical exam showed congestion, rhinorrhea, mucous membrane, and sternal retractions with increased work of breathing. Blood samples were drawn and a basic metabolic panel (BMP) showed an elevated anion gap at 24, indicative of the development of metabolic acidosis. A respiratory panel revealed a positive result for respiratory syncytial virus (RSV). A chest X-ray indicated minimal patchy airspace opacity in the right parahilar region, suggestive of early pneumonia, but no pleural effusion or pneumothorax was observed (Figure 1).

Due to the patient’s increased work of breathing, she was placed on supplemental oxygen via nasal cannula. A nebulized breathing treatment with albuterol and ipratropium bromide was administered. She was admitted for dehydration and was started on ½ normal saline/potassium chloride 20 mEq at 40 mL/hr. Her home medication regimen was also initiated to reduce the chance of seizures, along with acetaminophen suppository 160 mg. Due to the patient’s complications and increased risk of seizure, she was transferred for higher-level care the next day. She continued to have difficulty with oral intake, and weight loss and malnutrition became evident. Two days after transfer, a percutaneous endoscopic gastrostomy (PEG) was placed to help supplement her nutrition and fluid intake. She followed up with gastroenterology a week after the PEG insertion, and her overall well-being improved; she became more engaged, interactive, and communicative. With close follow-up, she was stable enough to tolerate solid foods and liquids orally and the PEG was removed within two months. The patient made a full recovery and was discharged home with her parents. Complications such as respiratory infections in a patient with DS can increase the complexity of care management for these patients.

## Discussion

Dravet syndrome (DS), previously known as severe myoclonic epilepsy of infancy, is a rare genetic disorder that is usually diagnosed within the first year of life. The hallmark of DS is its resistance to typical anti-epileptic medications, leading to treatment challenges and significant morbidity [10]. The management involves a multidisciplinary approach aimed at seizure control and optimizing developmental outcomes [10].

In young patients who develop early-life seizures, it is vital to illuminate the underlying cause of the seizure. Potential causes may include correctable factors such as electrolyte abnormalities or fever [10]. More serious conditions like DS must be considered as well; therefore, it is imperative to identify the root cause and address any reversible factors to mitigate the risk of further future complications. DS patients with concurrent viral infection causing fever are at an enhanced risk of febrile seizure due to an already reduced seizure threshold [11]. First-line antiepileptic drugs, in combination with a ketogenic diet and vagus nerve stimulation, are considered as adjunctive therapies to combat anti-epileptic resistance in DS patients [8].

In the case of our patient, it may have been best to administer acetaminophen as a trial precautionary measure to manage her low-grade fever on presentation, as this could help reduce the likelihood of complications associated with DS. She may have benefitted from a more timely administration of acetaminophen to mitigate the chance of seizures occurring [11]. Stronger anti-epileptic medications such as phenobarbital or benzodiazepines may have allowed for long term management of seizure prevention.

## Conclusions

Dravet syndrome (DS) involves the management of many aspects of the patient's healthcare. The education and involvement of the parents are critical to the proper care of a patient with DS to identify the root cause and address any reversible factors to mitigate the risk of further future complications. Respiratory infections can complicate management due to lowering a seizure threshold and an underlying metabolic acidosis. A combination of prophylactic seizure medications and a ketogenic diet allows for long-term management of seizure prevention.
